# Lysophosphatidic Acid Inhibits Apoptosis Induced by Cisplatin in Cervical Cancer Cells

**DOI:** 10.1155/2015/598386

**Published:** 2015-08-20

**Authors:** Yanxia Sui, Ya Yang, Ji Wang, Yi Li, Hongbing Ma, Hui Cai, Xiaoping Liu, Yong Zhang, Shufeng Wang, Zongfang Li, Xiaozhi Zhang, Jiansheng Wang, Rui Liu, Yanli Yan, Chaofan Xue, Xiaowei Shi, Li Tan, Juan Ren

**Affiliations:** ^1^Department of Radiotherapy Oncology, First Affiliated Hospital of Xi'an Jiaotong University, Xi'an, Shaanxi 710061, China; ^2^Pathology Department, First Affiliated Hospital of Xi'an Jiaotong University, Xi'an, Shaanxi 710061, China; ^3^Medical School, Xi'an Jiaotong University, Xi'an, Shaanxi 710061, China; ^4^ICU, China Meitan General Hospital, 29 Xi Ba He Nan Li, Chaoyang District, Beijing 100028, China; ^5^Department of Chemotherapy Oncology, First Affiliated Hospital of Xi'an Jiaotong University, Xi'an, Shaanxi 710061, China; ^6^Department of Oncology, Second Affiliated Hospital of Xi'an Jiaotong University, Xi'an, Shaanxi 710004, China; ^7^Department of Anesthesia, Xi'an Jiaotong University, Xi'an, Shaanxi 710061, China; ^8^Department of Orthopedics, Xi'an Children Hospital, Xi'an, Shaanxi 710000, China; ^9^Department of Surgery, First Affiliated Hospital of Xi'an Jiaotong University, Xi'an, Shaanxi 710061, China

## Abstract

Cervical cancer is the second most common cause of cancer death in women worldwide. Lysophosphatidic acid (LPA) level has been found significantly increased in the serum of patients with ovarian, cervical, and colon cancers. LPA level in cervical cancer patients is significantly higher than in healthy controls. LPA receptors were found highly expressed in cervical cancer cells, suggesting LPA may play a role in the development of cervical cancer. The aim of this study is to investigate the effect of LPA on the apoptosis induced by cisplatin (DDP) in cervical cancer cell line and the underlying changes in signaling pathways. Our study found that cisplatin induced apoptosis of Hela cell through inhibiting expression of Bcl-2, upregulating the expression of Bax, Fas-L, and the enzyme activity of caspase-3 (*p* < 0.05); LPA significantly provided protection against the apoptosis induced by cisplatin by inhibiting the above alterations in apoptotic factor caused by cisplatin (*p* < 0.05). Moreover, PI3K/AKT pathway was found to be important for the LPA antiapoptosis effect, and administration of PI3K/AKT partially reversed the LPA-mediated protection against cisplatin-induced apoptosis (*p* < 0.05). These findings have shed new lights on the LPA bioactivity in cervical cancer cells and pointed to a possible sensitization scheme through combined administration of PI3K inhibitor and cisplatin for better treatment of cervical cancer patients, especially those with elevated LPA levels.

## 1. Introduction

Cervical cancer is the second most common type, and the second most cause of deaths, of malignancy, in females worldwide. An estimated 500,000 new cases of cervical cancer are diagnosed, leading to 280,000 deaths, each year worldwide. The highest incidences of cervical cancers occur in developing countries. While surgery and chemoradiotherapy can cure 80%–95% of women with early stage cervical cancer, the recurrence and metastasis events are often associated with poor prognosis. In addition to the efforts for more effective prevention, new diagnosis and treatment modalities are urgently needed for better management of this life-threatening disease.

High levels of lysophosphatidic acid (LPA) were firstly found in the ascites of ovarian cancers patients [1, 2]. LPA is known as an “ovarian cancer activating factor” to exert a growth factor-like effect through binding to 4 specific G protein-coupled receptors (LPA1-4). The biological activities of LPA in ovarian cancer have been studied for many years. Increased level of lysophosphatidic acid is also found in patients with acute myocardial infarction. LPA has been implicated in the development of the cardiovascular system, assisting in its progression to a fully functional state [3, 4]. LPA is a bioactive glycerophospholipid generated and released by platelets, macrophages, epithelial cells, and tumor cells. LPA modulates a broad range of cellular responses, including alterations of cell proliferation, protection against apoptosis, modulation of chemotaxis, and transcellular migration [5, 6], thereby affecting the survival of ovarian cancer cells, macrophages, fibroblasts, and neonatal cardiac myocytes. The significant role of LPA in triggering these cellular responses has implicated LPA in tumor progression.

It has also been reported that LPA is increased in the plasma of cervical cancer patients [2]. Xu et al. found that stage I and stage IV cervical cancer patients had significantly higher plasma LPA levels than normal controls. Elevated LPA levels were detected in all the 6 cervical cancer patients examined [2]. In addition, there was an increased ratio of total LPA/lysophosphatidylinositol (LPI) [7]. Similarly, Shen et al. reported that the ratio of unsaturated LPA/LPI subspecies was significantly higher in patients with cervical cancer than in healthy controls [7]. The significantly increased LPA in the plasma of patients of cervical cancer points to its possible role(s) for the development of this malignancy. Indeed, LPA receptors were also found to be extensively expressed in cervical cell lines including Hela, CaSki, and Siha [8–11]. Previous studies from this group confirmed a high expression level of LPA receptors, especially the LPA receptor 2, in Hela cells [12], providing a basis for using this cell line as a study model to investigate the LPA bioactivity and the underlying pathways.

Cisplatin (DDP) has been used as the first line chemotherapy drug for adjuvant treatment of cervical cancer patients. Cisplatin-induced DNA damage activates multiple signaling pathways leading to cell apoptosis [13–15]. DNA damage caused by cisplatin induces the phosphorylation and stabilization of p53 [16]. p53 promotes cisplatin-induced apoptosis by antagonizing the antiapoptotic effect of Bcl-xL [17]. Phosphatidylinositol 3-kinase/AKT pathway is also involved in apoptosis regulation. Yan et al. found that suppression of PI3K/AKT pathway caused apoptosis in the HepG2 human hepatoma cell line [18]. On the other hand, Wang et al. found that LPA protects bone marrow-derived mesenchymal stem cells (BMSCs) against hypoxia- or serum deprivation-induced apoptosis [19]. LPA rescues H_2_O_2_-induced apoptosis by activating ERK1/2 and PI3K/AKT pathways in mesenchymal stem cells [19]. However, LPA effect on the apoptosis in cervical cancer cells and the potential mechanism remains unclear.

In this study we investigate how LPA-triggered cell responses may affect the cell apoptosis induced by cisplatin in a cervical cancer cell line. We characterized the effects of LPA on cell apoptotic factors including Bcl-2, Bax, and caspase-3 in Hela cells treated with cisplatin. The influence of LPA on the upstream pathway PI3K/AKT was also determined. The findings provide insights on the general bioactivity as well as the chemotherapeutic concerns of LPA produced by cervical cancer cells.

## 2. Materials and Methods

### 2.1. Materials

1-Oleoyl LPA (18 : 1 LPA) was purchased from Avanti Polar Lipids (Birmingham, AL, USA). Inhibitor of phosphoinositide 3-kinase (PI3K), LY290042, and inhibitor of mitogen-activated protein kinase (MAPK), PD98059, were from Cell Signaling (Beverly, MA, USA). Rho kinase inhibitor, Y-27632, was from Biomol (Beverly, MA, USA). Hela cells were cultured in Dulbecco's modified Eagle's medium (DMEM) supplemented with 100 mL/L fetal bovine serum (FBS), streptomycin (100 mg/L), and penicillin (100 KU/L) at 37°C in 50 mL/L CO_2_ incubator. Cells were serum starved for 12 hours before LPA treatment.

### 2.2. Cell Proliferation Assay

3-(4,5-Dimethylthiazol-2-yl)-2,5-diphenyltetrazolium bromide (MTT) colorimetry assay was employed to measure cell proliferation. Hela cells (2 × 10^3^/well) were seeded at 96-well plate. After being starved for 12 hours, cells were fed with DMEM containing LPA supplemented with 1 g/L bovine serum albumin. Following 24, 48, 72, and 96 hours of culture, 20 *μ*L of MTT solution (5 g/L) was added to each well. Four hours later, the medium was removed and 150 *μ*L of dimethyl sulfoxide (DMSO) was added to each well. Absorbance was measured at 490 nm on a Microplate Reader (EXL800). Each assay was performed in quintuplicate.

### 2.3. Annexin V Staining and Flow Cytometry Assay

Cells were starved in serum-free DMEM for 12 hours and treated with different concentrations of LPA. For kinase inhibitor experiments, LY294002 (50 *μ*mol/L), PD98059 (10 *μ*mol/L), and Y-27632 (10 *μ*mol/L) were applied to cells 30 min before addition of LPA. Cells were resuspended in binding buffer (10 mmol/L HEPES/NaOH, pH7.4, 140 mmol/L NaCl, 2.5 mmol/L CaCl_2_) and stained with 5 *μ*L of annexin-FITC and 5 mg/L propidium iodide (PI). Following washing, cells were analyzed with flow cytometry (FACS Calibur cytometer, BD Biosciences, USA) and CellQuest (BD Biosciences) for quantification of cell apoptosis. The experiment was performed in triplicate and repeated twice.

### 2.4. Caspase-3 Activity Assay

Activity of caspase-3 was detected using the CaspACE Assay System (Promega, USA). Briefly, cell lysates from treated and untreated control cells were prepared. Cell lysates were centrifuged at 15,000 ×g for 20 min at 4°C, and the supernatant was collected. The assay was performed in a total volume of 100 *μ*L in 96-well plates. Cell extracts with an equal amount of protein (50–100 *μ*g of total protein) were mixed with specific colorimetric substrate (DEVD-pNA) for caspase-3. The mixture was incubated at 37°C for 4 h according to the manufacturer's protocol. The absorbance was measured at a wave length of 405 nm.

### 2.5. Western Blotting

Antibodies against AKT (1 : 200 dilution times) and phosphorylated-AKT (1 : 200 dilution times) were purchased from Cell Signaling Transduction (USA). Antibodies against Fas (1 : 200 dilution times), Bcl-2 (1 : 200 dilution times), and Bax (1 : 200 dilution times) were purchased from Santa Cruz. After treatment, cells were rinsed with ice-cold PBS and then lysed in SDS sample buffer. Samples were resolved in electrophoresis using 10–12% sodium dodecyl sulfate (SDS) polyacrylamide gels and transferred to PVDF membranes (Bio-Rad, Hercules, CA). Immunoblot analyses were carried out using the appropriate antibodies. Specific proteins were detected with the enhanced chemiluminescence (ECL) system (Amersham Pharmacia Biotech, Piscataway, NJ).

### 2.6. Statistical Analysis

Statistical significance was assessed by one-way ANOVA using SPSS13.0 software. The Band in the Western blotting was quantified with software Quantity One. Data are presented as the means ± standard error. *p* ≤ 0.05 was used as standard for statistical significance.

## 3. Results

### 3.1. Effects of LPA on Proliferation of Hela Cell

**Figure 1:**
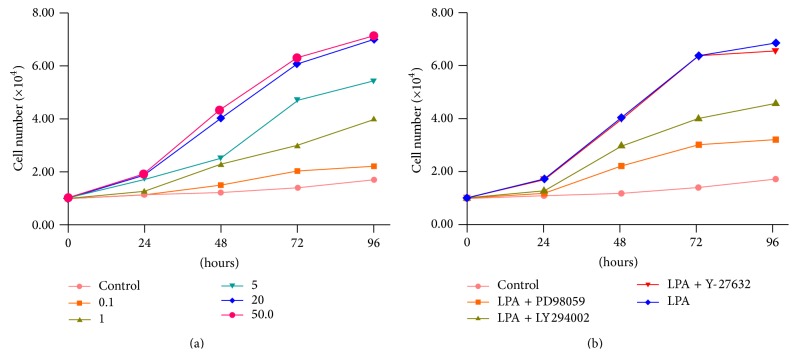
LPA effects on cell proliferation of Hela cells. (a) Hela cells were treated with different doses and time of LPA; MTT essay was performed. LPA significantly stimulated the proliferation of Hela cells in a dose-dependent and time-dependent manner. (b) Effect of inhibitors on LPA stimulation of cell proliferation. Hela cells were treated with LPA at 20 *μ*M plus different inhibitors for different time periods before MTT essay. PD98059 (MAPK inhibitor, at 10 *μ*M), PD98059 (MAPK inhibitor, at 10 *μ*M), and Y-27632 (Rho inhibitor, at 10 *μ*M) were applied, respectively. MAPK inhibitor, PD98059, significantly blocked the stimulation effect of LPA on cell proliferation in Hela cells. PI3K inhibitor, LY294002, partially blocked the LPA effect on cell proliferation.

Cells were treated with LPA at different concentrations including 0 *μ*M, 0.1 *μ*M, 1 *μ*M, 5 *μ*M, 20 *μ*M, and 50 *μ*M for different time periods including 24 hours, 48 hours, 72 hours, and 96 hours. It was found that LPA significantly stimulated the proliferation of Hela cells (*p* < 0.05). Between 0.1 *μ*M and 20 *μ*M, the higher the LPA concentration is, the greater the stimulation effect on cell proliferation was observed. There was no significant difference between the stimulation effect of 20 *μ*M LPA and 50 *μ*M LPA on cell proliferation. The stimulation effect of LPA on proliferation of Hela cells was also increased along with the time. The longer the exposure time, the greater the stimulation effect of LPA (Figure 1(a)).

Several inhibitors were employed to determine which signaling transduction pathway was involved in the LPA effect on Hela cell proliferation. The inhibitors include LY294002 (PI3K inhibitor), PD98059 (MAPKinase inhibitor), and Y-27632 (Rho kinase inhibitor). Cells were treated with 20 *μ*M of LPA plus different inhibitors. It was found that PD98059 (MEK1 inhibitor) significantly blocked the stimulation effect of LPA on the proliferation of Hela cell (*p* < 0.05). LY294002 (PI3K inhibitor) also partially blocked the stimulation effect of LPA on the proliferation of Hela cell (Figure 1(b)). The results suggested that Ras/Raf-MAPK signal pathway may be involved in the LPA stimulation of Hela cells proliferation.

### 3.2. Effect of LPA on Apoptosis Induced by Cisplatin

**Figure 2:**
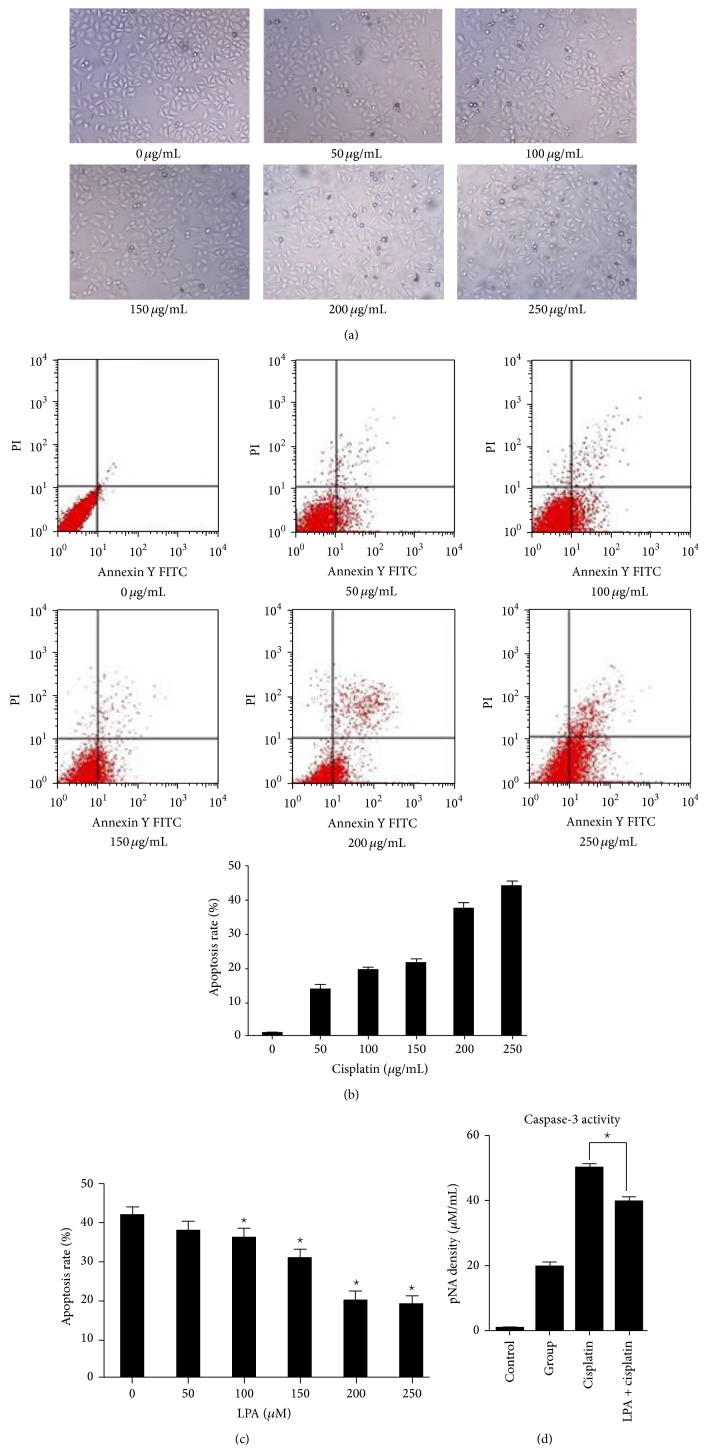
LPA effects on apoptosis induced by cisplatin in Hela cell. (a) Cell pictures (×20). Hela cells were treated with cisplatin at different doses. After 8 hours of treatment with cisplatin, many floating cells were observed. The bigger the dose of cisplatin, the more the floating cells. (b) Flow cytometry detection of apoptosis with Annexin V/PI staining. Hela cells were treated with cisplatin at different doses for 4 hours. Apoptotic cells were detected and quantified with flow cytometry. Increased apoptosis following cisplatin treatment was observed. (c) Quantification of LPA effects on apoptosis induced by cisplatin. Hela cells were treated with cisplatin (at 200 *μ*g/mL) and LPA (at different concentrations) for 4 hours. The apoptotic cells were detected by flow cytometry and the apoptosis rate of Hela cells was quantified and shown. Cell apoptosis induced by cisplatin was significantly inhibited by LPA. (d) Caspase-3 activity. Hela cells were treated with 200 *μ*g/mL of cisplatin plus LPA at 20 *μ*M for 4 hours. The caspase-3 activity was measured and shown. Cisplatin dramatically increased the caspase-3 activity, but the upregulated activity of caspase-3 was partially reversed by LPA treatment.

Following treatment with increasing concentrations of cisplatin (50–250 *μ*g/mL) for 8 hours, many floating cells were observed (Figure 2(a)), indicating the occurrence of extensive cell deaths. To confirm the cells died of apoptosis, Hela cells treated with cisplatin were subject to flow cytometry analysis and the assay of caspase-3 activity. Similar levels of apoptosis were detected by flow cytometry and measurement of caspase-3 activity. The apoptosis rates elevated with the increase of cisplatin concentrations. The apoptosis rate was 0.96 ± 0.07%, 13.65 ± 1.36%, 19.49 ± 0.75%, 21.44 ± 1.16%, 37.48 ± 1.65%, and 44.15 ± 1.33% when cisplatin was applied at 50, 100, 150, 200, and 250 *μ*g/mL, respectively (Figure 2(b)).

To examine the LPA effects on cisplatin-induced apoptosis, Hela cells were treated by 200 *μ*g/mL cisplatin alone or plus LPA for 4 hours. It was found that apoptosis induced by cisplatin was significantly inhibited by LPA. Moreover, this inhibition displayed a dose-dependency. The inhibition for cell apoptosis became significant (*p* < 0.05) when the concentration of LPA was ≥20 *μ*M (Figure 2(c)).

In caspase-3 activity assay, Hela cells were treated with 200 *μ*g/mL of cisplatin alone or plus 20 *μ*M of LPA for 4 hours. It was found that cisplatin treatment significantly increased the caspase-3 activity, but the upregulation of caspase-3 activity was partially reversed by LPA (Figure 2(d)).

### 3.3. LPA Effects on the Expression of Apoptosis Proteins

**Figure 3:**
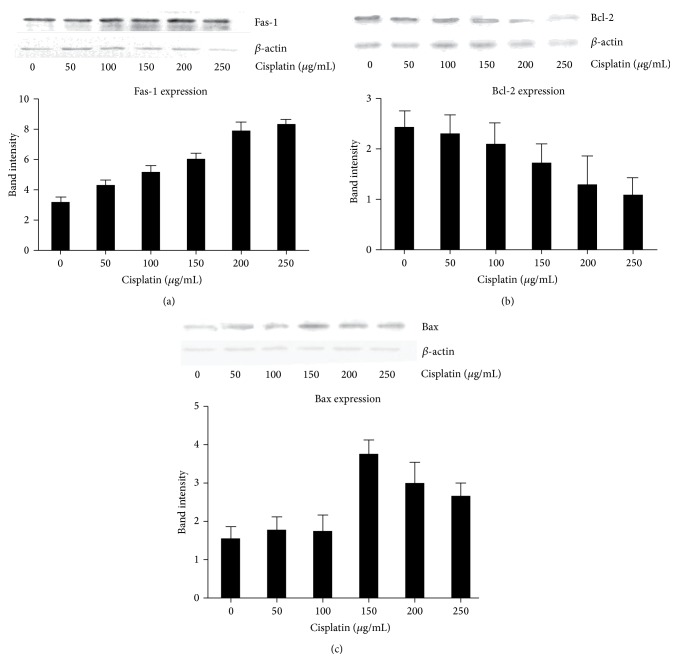
Western blotting analysis of apoptotic proteins in cells treated with cisplatin. Hela cells were treated with cisplatin at different doses including 0.50 *μ*g/mL, 100 *μ*g/mL, 150 *μ*g/mL, 200 *μ*g/mL, and 250 *μ*g/mL, for 4 hours. *β*-actin was used as a protein loading control. The resultant bands were subject to densitometry analysis for quantification. (a) Western blotting of Fas-L. The expression of Fas-L was significantly induced by cisplatin in a dose-dependent manner. (b) Western blotting of Bcl-2. The expression of Bcl-2 was significantly inhibited by cisplatin in a dose-dependent manner. (c) Western blotting of Bax. The expression of Bax was significantly induced by cisplatin in a dose-dependent manner.

**Figure 4:**
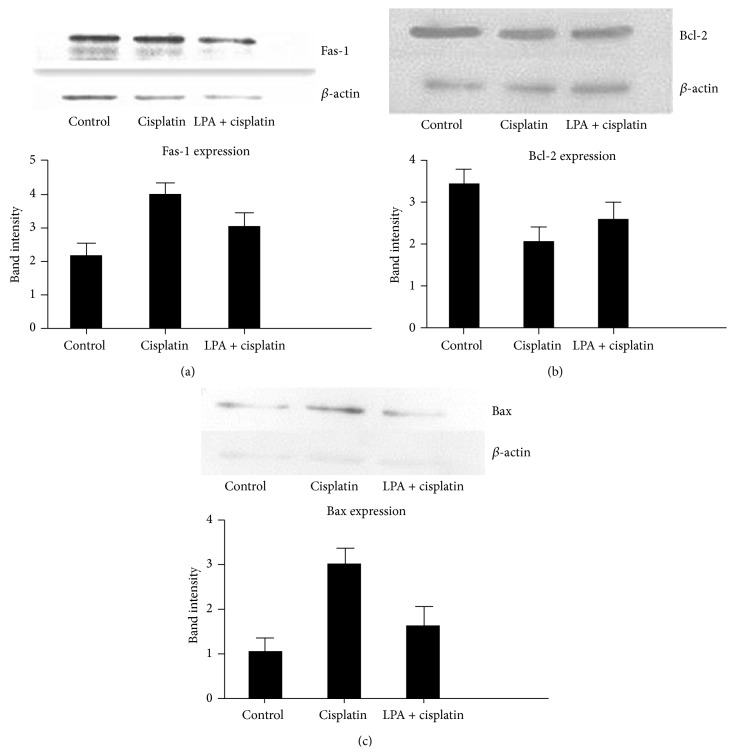
LPA effects on the expression of apoptotic proteins induced by cisplatin. Hela cells were treated with cisplatin (at 200 *μ*g/mL) alone or plus LPA (at 20 *μ*M) for 4 hours. Western blotting was used to determine the expression of apoptosis related proteins. *β*-actin was used as a protein loading control. (a) Western blotting of Fas-L. The increased Fas-L expression induced by cisplatin was significantly reversed by LPA treatment. (b) Western blotting of Bcl-2. LPA restored the Bcl-2 expression which was inhibited by cisplatin. (c) Western blotting of Bax. The upregulation of Bax expression induced by cisplatin was significantly inhibited by LPA.

Since cisplatin induced apoptosis in cervical cancer Hela cell, we further characterized alterations of key proteins involved in the apoptotic pathway. Western blotting showed that the expression of Fas-1 was significantly increased by cisplatin treatment in a dose-dependent manner (Figure 3(a)). Expression of the antiapoptotic protein, Bcl-2, was significantly inhibited by cisplatin. Moreover, the inhibition of Bcl-2 expression was dependent on the dose of cisplatin (Figure 3(b)). Expression of another important apoptotic protein, Bax, was also significantly increased by cisplatin treatment (Figure 3(c)). Since LPA appeared to rescue Hela cells from apoptosis induced by cisplatin, we examined the LPA effect on the change of apoptosis-related proteins induced by cisplatin. Western blotting showed that the increase of Fas-1 expression induced by cisplatin was significantly inhibited by LPA (Figure 4(a)). Moreover, LPA treatment restored the inhibition of Bcl-2 expression by cisplatin (Figure 4(b)) in comparison to cells treated with cisplatin alone (Figure 2(b)). In addition, LPA significantly inhibited the increased Bax expression induced by cisplatin. The increase of Bax expression induced by cisplatin was significantly inhibited by LPA (Figure 4(c)). These results indicated that LPA was able to block the apoptotic effects of cisplatin by reversing the changes in apoptotic proteins caused by cisplatin treatment.

### 3.4. Signaling Transduction Pathways Involved in the LPA Inhibition of Apoptosis Induced by Cisplatin

**Figure 5:**
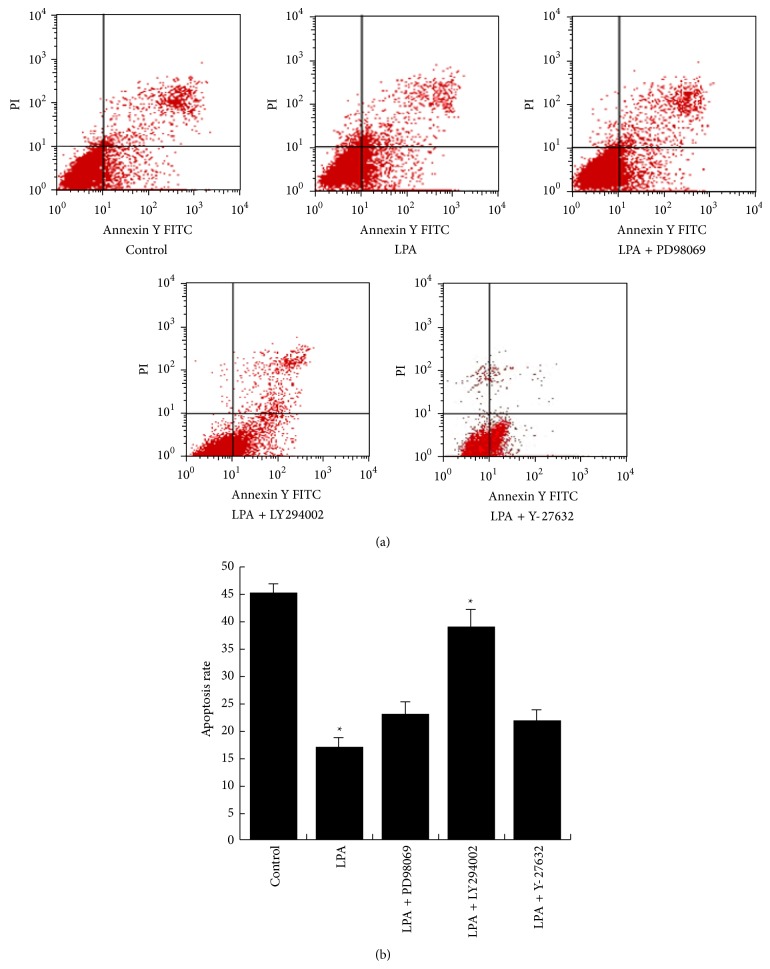
Flow cytometry detection of cell apoptosis by Annexin V/PI staining. (a) Hela cells were treated with cisplatin (200 *μ*g/mL) and LPA (20 *μ*M), alone or in combination with different inhibitors for 4 hours. Cisplatin and LPA were used in each inhibitors-treated group. Inhibitors including PD98059 (MAPK inhibitor, at 10 *μ*M), PD98059 (MAPK inhibitor, at 10 *μ*M), and Y-27632 (Rho inhibitor, at 10 *μ*M) were applied, respectively. (b) The apoptosis rate of Hela cells was quantified. Cisplatin-treated group served as control. PI3K inhibitor LY294002 significantly reversed the LPA effect on apoptosis induced by cisplatin, while MAPK inhibitor PD98059 and Rho inhibitor Y-27632 had no significant effect.

Since several studies have shown cell apoptosis is associated with the activation of PI3K/AKT, Ras/MAPK, and RHO/GEFs/RHOA pathways, we applied specific inhibitors specifically targeting these pathways to elucidate the mechanism underlying the LPA antiapoptotic activity. Cells were treated by 200 *μ*g/mL cisplatin alone or plus 20 *μ*M LPA and, additionally, different inhibitors including PD98059 (MAPK inhibitor, at 10 *μ*M), LY294002 (PI3K inhibitor, at 10 *μ*M), or Y-27632 (Rho inhibitor, at 10 *μ*M). It was found that the PI3K inhibitor LY294002 significantly reversed the protection effect of LPA on apoptosis induced by cisplatin (*p* = 0.003), while MAPK inhibitor PD98059 and Rho inhibitor Y-27632 had no significant effect (Figures 5(a) and 5(b)). These results suggested that the LPA effect on apoptosis induced by cisplatin were likely mediated by the PI3K/AKT signaling pathway, but not Ras/MAPK and RHO/GEFs/RHOA pathways.

### 3.5. Effects of LPA on the Phosphorylation of AKT

**Figure 6:**
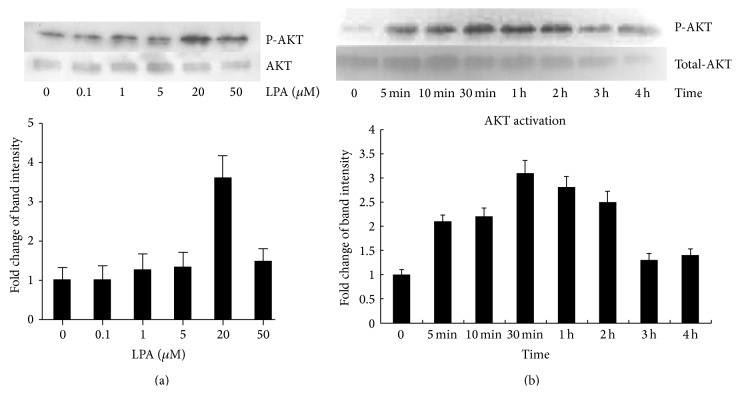
Western blotting analysis of phosphorylated AKT in cells treated with LPA. (a) Hela cells were treated with LPA at different concentrations including 0, 0.1 *μ*M, 1 *μ*M, 5 *μ*M, 20 *μ*M, and 50 *μ*M for 1 hour. Western blotting was conducted to detect the expression of phosphorylated AKT. Total AKT was detected and the results were used as protein input control. Densitometry analysis was carried out for quantification purpose. LPA treatment led to increased phosphorylation of AKT in a dose-dependent manner. (b) Time-response effect of LPA on the phosphorylation of AKT. Hela cells were treated with LPA at 20 *μ*M for different times including 5 min, 10 min, 30 min, 1 hour, 2 hours, 3 hours, and 4 hours. Western blotting was used to detect the expression of phosphorylated AKT. Compared to the total AKT, LPA treatment activated the phosphorylation of AKT in a time-dependent manner.

Since AKT inhibitor significantly reversed the LPA protection of cell apoptosis caused by cisplatin, we subsequently investigated if LPA could activate the PI3K/AKT pathway by increasing the phosphorylation of AKT. Cells were treated by LPA at different concentrations including 0 *μ*M, 0.1 *μ*M, 1 *μ*M, 5 *μ*M, 20 *μ*M, and 50 *μ*M of LPA for 1 hour (Figure 6(a)). The results showed that LPA treatment led to an increased phosphorylation of AKT in a dose-dependent manner. In addition, LPA-mediated activation of AKT was also characterized by the time-dependent manner. When cells were treated with 20 *μ*M of LPA for different time, AKT phosphorylation gradually increased, reaching the peak at the 2-hour time point. There was a significant difference between control and the LPA-treated groups at 5 min, 10 min, 30 min, 1 hour, and 2 hours (Figure 6(b)). These results indicated that LPA protection effect on the apoptosis induced by cisplatin may be related to the phosphorylation of AKT.

### 3.6. Effects of LPA on the Phosphorylation of ERK

**Figure 7:**
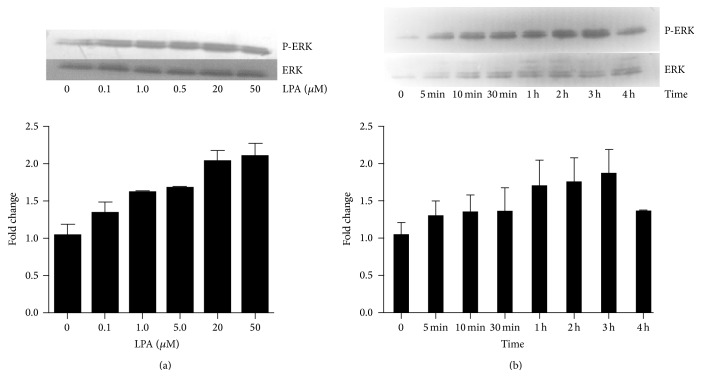
Western blotting analysis of phosphorylated ERK in cells treated with LPA. (a) Hela cells were treated with LPA at different concentrations including 0, 0.1 *μ*M, 1 *μ*M, 5 *μ*M, 20 *μ*M, and 50 *μ*M, for 1 hour. Western blotting was performed to detect the levels of phosphorylated ERK. Total ERK was used as protein input control. The density of bands was quantified and compared. LPA activated the phosphorylation of ERK in a dose-dependent manner. (b) Hela cells were treated with LPA at 20 *μ*M for different times including 5 min, 10 min, 30 min, 1 hour, 2 hours, 3 hours, and 4 hours. A time-dependent increase in the ERK phosphorylation was detected.

Since MAPK inhibitor, PD98059, significantly inhibited the proliferation stimulated by LPA, which suggested ERK/MEK/MAPK pathway was involved in the LPA functions in the proliferation in Hela cells, we investigated whether LPA can activate the phosphorylation of ERK. The results showed that LPA significantly activated the phosphorylation of ERK in a dose-dependent manner. Cells were treated with LPA at different concentrations including 0 *μ*M, 0.1 *μ*M, 1 *μ*M, 5 *μ*M, 20 *μ*M, and 50 *μ*M for 1 hour. The effect on ERK phosphorylation of LPA was increased along with the increase of LPA concentration (Figure 7(a)).

LPA also activated ERK in a time-dependent manner. Cells were treated by 20 *μ*M LPA at different time points including 0 min, 5 min, 10 min, 30 min, 1 hour, 2 hours, 3 hours, and 4 hours. ERK phosphorylation reached the peak at 3 hours following LPA treatment (Figure 7(b)).

## 4. Discussion

In the intrinsic or mitochondrial pathway of apoptosis, Bcl-2 is able to prevent cell apoptosis, while a related protein, Bax, can migrate to the surface of the mitochondrion where it inhibits the protective effect of Bcl-2 by interrupting the membrane structure, causing the release of cytochrome C and caspase activation. In the extrinsic or death receptor pathway of apoptosis, Fas and the TNF receptor are integral membrane proteins receiving apoptotic signals. Upon binding by ligand, they transmit signals to the cytoplasm, which leads to activation of caspase and phagocytosis.

Some studies found that cell apoptosis induced by cisplatin was mediated by death receptors signaling pathway [20, 21], while others claimed that it was mediated by mitochondrial pathway [22, 23]. The discrepancy in previous studies may be caused by the divergent cell lines, methodologies, and conditions used by different groups. In this study, we observed that cisplatin significantly induced the apoptosis of Hela cells in a concentration-dependent manner. Cisplatin treatment was accompanied by increased Fas-1 and Bax expression and the increased enzyme activity of caspase-3. Meanwhile cisplatin inhibited Bcl-2 expression. These observations suggested that cisplatin-induced apoptosis of Hela cell may be mediated by both intrinsic and extrinsic pathways. Indeed, extensive cross talks between the two pathways have been identified [24, 25]. Simultaneous activation of the two pathways has been observed in many types of cells following various treatments.

Our previous study found that both ovarian cancer cells and peritoneal mesothelial cells contribute to the elevated LPA levels in ovarian cancer ascites. Under normal culture conditions, ovarian cancer cells and prostate cancer cells, but not breast cancer cell lines, constitutively synthesize and release significant amounts of LPA to the culture medium. A major finding of this study is that LPA was able to rescue the Hela cells apoptosis induced by cisplatin. The protective role of LPA against apoptosis was observed in ovarian cancer cells [26]. We observed that LPA treatment blocked the alterations of Bcl-2, Fas-1, and Bax expression that were induced by cisplatin. LPA also significantly inhibited the upregulation of the caspase-3 enzyme activity induced by cisplatin. Our observations of LPA effect on cervical cancer were consistent with other reports on LPA effects in different kinds of cells. Meng et al. found that LPA may protect epithelial ovarian cancer from immune cell attack and cytoskeleton disrupting reagents induced apoptosis through multiple pathways and LPA inhibited anti-Fas-induced apoptosis enhanced by actin depolymerization [26, 27]. Actin depolymerization accelerated caspase-8 activation, while LPA inhibited the association and activation of caspase-8 at the DISC in epithelial ovarian cancer OVCAR3 cells. Thus, our observations have raised an interesting question on the potential role of LPA for the development of chemoresistance by cervical cancer cells. In addition, could LPA be an effective biomarker for the prediction of chemoresistance in cervical cancer? Further studies are required to answer these questions.

In this study, three specific kinase inhibitors were applied to determine the signaling pathways involved in the protection effect of LPA on apoptosis. It was found that PI3K inhibitor (LY290042) almost completely blocked the LPA protection against apoptosis induced by cisplatin. Subsequent experiments confirmed that LPA treatment of Hela cells led to increased phosphorylation of AKT in a dose- and time-dependent manner. This indicated that LPA protected Hela cells from apoptosis induced by cisplatin. This effect of LPA may be through the activation of PI3K/AKT pathway. This finding appears to be consistent with published data showing that PI3K/AKT pathway plays a key role in the regulation of cell apoptosis [28, 29]. Wan et al. observed that PI3K inhibition augmented staurosporine-induced apoptosis in the endometrial carcinoma cell line [28]. In that experiment, staurosporine reduced the levels of phosphorylation in AKT and Bad; In a study by Kim et al. 2,4-bis(p-hydroxyphenyl)-2-butenal (HPB242) induced apoptosis via the inhibition of PI3K/AKT pathway in human cervical cancer cells. HPB242 treatment decreased phosphatidyl inositol 3-kinase and p-AKT expression levels, demonstrating that this survival pathway may also be inhibited by HPB242 [30]; In another study, Cui et al. found that MiR-125b inhibited tumor growth and promoted apoptosis of cervical cancer cells by targeting phosphoinositide 3-kinase catalytic subunit delta. Overexpression of MiR-125b in Hela cervical cancer cells induced apoptosis and downregulated the expression of PIK3CD and Phospho-AKT [31]; Kang et al. found that thioridazine induces apoptosis by targeting the PI3K/AKT/mTOR pathway in cervical cancer cells [32]; Thanaketpaisarn et al. found that Artesunate enhanced TRAIL-induced apoptosis in human cervical carcinoma cells through inhibition of the PI3K/AKT and NF-*κ*B signaling pathways [33]; Wang et al. found that Stathmin is involved in arsenic trioxide-induced apoptosis in human cervical cancer cell lines via PI3K linked signal pathway. As2O3-induced stathmin downregulation is mediated through the phosphatidylinositol-3-kinase (PI3K) signaling pathway. PI3K inhibitor effectively attenuated the stathmin downregulation and cell apoptosis upon As2O3-treatment [34]; Recurrent and metastatic cervical cancers often acquire chemoresistance to cisplatin. Our finding pointed to a possible sensitization scheme through combined administration of PI3K inhibitor and cisplatin.

It should be pointed out that while our current study focused on the apoptotic pathways and mechanisms, the LPA effects on cell cycle control and cell proliferation should not be excluded. In fact the PI3K/AKT pathway is known to play an active role in the control of cell cycle [35]. We found that MEK1 inhibitor (PD98059) significantly inhibited the effect of LPA on the proliferation of Hela cells. Ras/Raf1/MEK/ERK signal transduction pathway is one of the most important members of the mitogen-activated protein kinase pathways regulating cell proliferation [36]. MEK1, a MAPK, represents a key molecule of the Ras/Raf1/MEK/ERK signal pathway [37]. Several lines of evidence suggest that, in cervical cancer, the Ras/Raf1/MEK/ERK pathway, but not the JNK pathway or the p38 MAPK pathway, is the major regulator of cell proliferation [38–40].

In summary, we demonstrated that cisplatin induced apoptosis of Hela cell via the downregulation of Bcl-2 and upregulation of Bax, Fas-L, and increased enzyme activity of caspase-3. LPA was able to block the alterations in the apoptotic factors caused by cisplatin and, ultimately, reversed the apoptosis induced by cisplatin. Cisplatin-induced cell apoptosis appeared to be partially dependent on PI3K/AKT pathway, and AKT inhibitor was able to reverse the protective effects of LPA against cisplatin-induced apoptosis. LPA stimulated the proliferation of Hela cells through the Ras/Raf1/MEK/ERK pathway. These data enriched our knowledge regarding the LPA activity in cervical cancer cells. In addition, the finding on the positive impact of PI3K/AKT inhibitor on the apoptotic effects of cisplatin has provided useful information for the design of sensitization strategy in cervical cancer patients, especially those with high levels of LPA.
